# Baicalein alleviates fibrosis and inflammation in systemic sclerosis by regulating B-cell abnormalities

**DOI:** 10.1186/s12906-023-03885-1

**Published:** 2023-02-21

**Authors:** Bo Peng, Qin Hu, Rong He, Hongping Hou, Dongyin Lian, Ying Chen, Han Li, Ling Song, Yunhang Gao, Tengfei Chen, Guangping Zhang, Jianrong Li

**Affiliations:** 1 Institute of Chinese Materia Medica, China Academy of Chinese Medical Sciences, Beijing, 100700 People’s Republic of China; 2 College of Life Sciences and Bio-Engineering, Beijing University of Technology, Beijing, 100024 People’s Republic of China

**Keywords:** Baicalein, Scleroderma, B cell, Autoimmunity, Fibrosis, Inflammation

## Abstract

**Background:**

Systemic sclerosis (SSc; also known as “scleroderma”) is an autoimmune disorder characterized by extensive fibrosis, vascular changes, and immunologic dysregulation. Baicalein (phenolic flavonoid derived from *Scutellaria baicalensis* Georgi) has been used to treat the pathological processes of various fibrotic and inflammatory diseases. In this study, we investigated the effect of baicalein on the major pathologic characteristics of SSc: fibrosis, B-cell abnormalities, and inflammation.

**Methods:**

The effect of baicalein on collagen accumulation and expression of fibrogenic markers in human dermal fibroblasts were analyzed. SSc mice were produced by injecting bleomycin and treated with baicalein (25, 50, or 100 mg/kg). The antifibrotic features of baicalein and its mechanisms were investigated by histologic examination, hydroxyproline assay, enzyme-linked immunosorbent assay, western blotting and flow cytometry.

**Results:**

Baicalein (5–120 μM) significantly inhibited the accumulation of the extracellular matrix and fibroblast activation in transforming growth factor (TGF)-β1- and platelet derived growth factor (PDGF)-induced human dermal fibroblasts, as evidenced by abrogated deposition of total collagen, decreased secretion of soluble collagen, reduced collagen contraction capability and downregulation of various fibrogenesis molecules. In a bleomycin-induced model of dermal fibrosis in mice, baicalein (25–100 mg/kg) restored dermal architecture, ameliorated inflammatory infiltrates, and attenuated dermal thickness and collagen accumulation in a dose-dependent manner. According to flow cytometry, baicalein reduced the proportion of B cells (B220^+^ lymphocytes) and increased the proportion of memory B cells (B220^+^CD27^+^ lymphocytes) in the spleens of bleomycin-induced mice. Baicalein treatment potently attenuated serum levels of cytokines (interleukin (IL)-1β, IL-2, IL-4, IL-6, IL-17A, tumor necrosis factor-α), chemokines (monocyte chemoattractant protein-1, macrophage inflammatory protein-1 beta) and autoantibodies (anti-scleroderma 70 (Scl-70), anti-polymyositis-scleroderma (PM-Scl), anti-centromeres, anti-double stranded DNA (dsDNA). In addition, baicalein treatment can significantly inhibit the activation of TGF-β1 signaling in dermal fibroblasts and bleomycin-induce mice of SSc, evidenced by reducing the expression of TGF-β1 and IL-11, as well as inhibiting both small mother against decapentaplegic homolog 3 (SMAD3) and extracellular signal-related kinase (ERK) activation.

**Conclusions:**

These findings suggest that baicalein has therapeutic potential against SSc, exerting modulating B-cell abnormalities, anti-inflammatory effects, and antifibrosis.

**Supplementary Information:**

The online version contains supplementary material available at 10.1186/s12906-023-03885-1.

## Introduction

Systemic sclerosis (SSc; also known as “scleroderma”) is an autoimmune disorder characterized by extensive fibrosis, vascular and inflammatory alterations, as well as the release of autoantibodies against nuclear/cellular antigens. SSc involves the skin, lungs, heart, gastrointestinal tract, and kidneys [1]. It is an incurable disease with a mean duration of 11.7 years [2]. SSc has the highest case-specific mortality among autoimmune diseases, with 50% of patients dying as a direct consequence of the disease [3]. Despite evidence of improved overall survival and treatment options, obtaining efficacious disease-modifying treatment for SSc is a serious  clinical challenge. Therefore, identification of promising therapeutic agents for SSc is needed urgently [4].

Baicalein is a primary phenolic flavonoid derived from the Chinese herb *Scutellaria baicalensis* Georgi. It possesses anticancer [5], anti-inflammatory, antioxidant [6], anxiolytic, and anti-infection effects [7]. Baicalein has been used increasingly against pulmonary, hepatic, cardiac and renal fibrosis due to its modulation of pro-fibrotic and proinflammatory pathways [8–11]. Fibrosis suppression by baicalein can be attributed (at least in part) to the attenuation of production of collagen and the extracellular matrix (ECM) [9, 12–14], inhibition of cell proliferation and induction of apoptosis of fibroblasts [15], inhibition of myofibroblast differentiation [8], and downregulation of expression of fibrogenic markers [9, 12, 14]. Moreover, baicalein was able to ameliorate the severity of different experimental autoimmune diseases, such as encephalomyelitis, collagen-induced arthritis and autoimmune hepatitis, which was indicated previously by reducing the production of proinflammatory cytokines/chemokines, promoting apoptosis in cluster of differentiation (CD)19^+^ splenocytes [16], suppressing pathogenetic CXCR6^+^ CD4 cells [17], inhibiting T cell proliferation via the JAK-STAT signaling pathway [18]. However, the effect of baicalein against SSc and the exact molecular mechanisms responsible for its effects have not been elucidated.

SSc is dominated by immunologic abnormalities, which implicate infiltration and activation of multiple immune cells followed by autoimmunity, secretion of proinflammatory cytokines, and chemokine release [19]. Continuously activated immune cells cause endothelial cell activation and an increase in the number of adhesion molecules to recruit immune cells [20]. Furthermore, activation of immune cells initiates the expression of profibrotic markers, activates fibroblasts, and transforms myofibroblasts [19]. B cells are a heterogeneous population of immune cells with different subsets. B cells play important roles in the fibrotic pathogenesis of SSc, as highlighted by autoantibody synthesis, production of proinflammatory or profibrotic cytokines, increased serum levels of immunoglobulin (Ig)G, inflammatory infiltration and interaction with other cells in the dermis [21]. Mounting evidence has suggested the phenotypical changes of B cells and B cell signaling aberrations in SSc [4, 21]. The frequency and the absolute number of B cells were increased in SSc [22], with approximately 20% overexpression of CD19 [4]. The unbalanced B cell subsets with a reduced number of memory B cells (especially “pre-switched” memory B cells) and switched memory B cells have been described in SSc patients in recent studies [23–25]. Some studies have confirmed the loss of memory B cells in SSc, which reported the augmented spontaneous apoptosis of CD27 + memory B cells, upregulation of the apoptosis regulator CD95 (Fas) [22], and downregulation of the antiapoptotic regulator B-cell lymphoma 2 (Bcl-2) [23] in subsets of memory B cells. These immunological dysregulations result in disrupted B cell homeostasis and favor the development of autoimmune reactions in SSc. Thus, B cells could be attractive therapeutic targets for SSc, which may lead to depletion or inhibition of critical cytokines, reduced production of autoantibodies by B-cell signaling, and deactivated fibroblasts by pro-fibrotic pathways [4]. Recent studies have shown the relationship between baicalein and immune cells to counteract fibrosis, such as the downregulation of expression of proinflammatory cytokines and chemokines (e.g., interleukin (IL)-1β, IL-6, tumor necrosis factor-alpha (TNF-α) and monocyte chemoattractant protein-1 (MCP-1)) [13] and inhibition of infiltration of inflammatory cells (macrophages and lymphocytes) [26]. Baicalein has been identified as an inhibitor of Lyn kinase [27], which is the first signal-transducing kinase required for B cell activation in SSc patients [28] and contributes to the development of systemic autoimmunity and skin sclerosis through CD19-dependent pathway in tight skin mice [29]. Moreover, spleen tyrosine kinase (Syk) is another tyrosine kinase after B cell receptor (BCR) activation which is involved in the sclerodermatous process via B cell signaling [30]. Thirty μM baicalein showed 23.1% inhibition on Syk kinase activity, which indicated that Syk may be the target molecule of baicalein [31]. Previous observations led us to postulate that baicalein might modulate B-cell abnormalities and be efficacious against SSc.

We aimed to investigate the effect of baicalein on the major pathologic characteristics of SSc in vitro and in vivo using a bleomycin-induced model of skin fibrosis in mice, including fibrosis, B-cell abnormalities, and inflammation.

## Materials and methods

### Cell line and culture

Normal human embryonic dermal fibroblasts (CCC-ESF-1) were obtained from the Cell Resource Center of Peking Union Medical College (Beijing, China). Cells were used between passages 10 and 14. Cells were maintained in Dulbecco’s modified Eagle’s medium supplemented with 10% fetal bovine serum (Gibco, Grand Island, NY, USA) in a humidified atmosphere of 5% CO_2_ at 37 °C.

### Drugs

Baicalein (catalog number: 111595–201607) was obtained from the National Institutes for Food and Drug Control (Beijing, China). A stock solution of baicalein (20 mM) was created in dimethyl sulfoxide. Working concentrations were prepared by diluting the stock solution in medium. Transforming growth factor-beta 1 (TGF)-β1 and platelet derived growth factor (PDGF) were obtained from Peprotech (Rocky Hill, NJ, USA). An inhibitor of TGF-β receptor type I (LY364947) and a selective inhibitor of PDGFRα/β (crenolanib) were obtained from MedChemExpress (Monmouth Junction, NJ, USA).

### Measurement of the accumulation and secretion of collagen

CCC-ESF-1 cells were seeded in collagen I-coated plates to create an in vitro model of fibrosis, and cultured for 48 h to 80–100% confluence. After washing with phosphate-buffered saline (PBS), the medium was changed to serum-free Dulbecco’s modified Eagle’s medium supplemented with 1% insulin–transferrin–selenium (the basic medium used throughout this study) for 24 h. Then, cells were incubated in the medium with or without TGF-β1 (5 ng/mL) or PDGF (40 ng/mL) for an additional 48 h in the presence or absence of different concentrations of baicalein. Some inhibitors (LY364947 (1 μM) and crenolanib (2 μM)) were used as positive controls. Collagen deposition was assessed by picrosirius red (PSR) staining. Cells were fixed with methanol overnight at − 20 °C and incubated in 0.1% PSR staining solution for 4 h. Then, cells were washed twice with 0.1% acetic acid and PSR was eluted in 0.1 M sodium hydroxide. The optical density (OD) was measured at 540 nm. Collagen secretion was detected by measuring soluble collagen in cell-culture supernatants using the Sircol Soluble Collagen Assay (Biocolor, Country Antrim, UK) according to manufacturer instructions.

### Cytotoxicity assay

Cytotoxicity was assessed using the lactate dehydrogenase (LDH) assay. The supernatant (120 μL) from each well was collected. LDH release was measured according to the instructions of the manufacturer (Beyotime Institute of Biotechnology, China). Cytotoxicity was determined according to the OD of LDH release by cells.

### Enzyme-linked immunosorbent assays (ELISAs)

CCC-ESF-1 cells were lysed with three freeze–thaw cycles between − 80 °C and 37 °C in PBS and centrifuged at 12,000 × *g* for 15 min at 4 °C. Levels of type-I collagen, α-smooth muscle actin (α-SMA) and IL-11 in supernatants were measured using ELISA kits according to manufacturer (Cusabio, Beijing, China) instructions.

### Real-time reverse transcription-quantitative polymerase chain reaction (RT-qPCR)

Total RNA in cells was isolated by the RNeasy Mini kit (Qiagen, Hilden, Germany). Complementary (c)DNA was prepared from 2 μg of total RNA using the RevertAid™ First Strand cDNA Synthesis Kit (Thermo Scientific, Waltham, MA, USA). Then, cDNA was subjected to RT-qPCR to quantify gene expression using SYBR Green SuperReal Premix Plus (Tiangen, Beijing, China). The primer pairs used for analyses are listed in Table 1. Glyceraldehyde 3-phosphate dehydrogenase was used to normalize the amounts of loaded cDNA. Relative expression of target genes was calculated using the 2^−ΔΔCt^ method.

**Table 1:** Primer sequences used in this study

Human genes	Primer sequence	Length (bp)
Collagen type I alpha 1 chain (COL1A1)	Forward: 5′-TCAAGAGAAGGCTCACGATGG-3′ Reverse: 5′-TCACGGTCACGAACCACATT-3′	69
Collagen type I alpha 2 chain (COL1A2),	Forward: 5′-GGTCAGCACCACCGATGTC-3′ Reverse: 5′- CACGCCTGCCCTTCCTT-3′	51
Collagen type III alpha 1 chain (COL3A1),	Forward: 5′- GCTGGCTACTTCTCGCTCTG-3′ Reverse: 5′- TCCGCATAGGACTGACCAAG-3′	97
Cellular communication network factor 2 (CCN2)	Forward: 5′- CAGCATGGACGTTCGTCTG-3′ Reverse: 5′- AACCACGGTTTGGTCCTTGG-3′	115
Fibronectin 1 (FN1)	Forward: 5′- TTCTAAGATTTGGTTTGGGATCAAT-3′ Reverse: 5′- TCTTGGTTGGCTGCATATGC-3′	51
Transforming growth factor beta 1 (TGFB1)	Forward: 5′- CAATTCCTGGCGATACCTCAG-3′ Reverse: 5′- GCACAACTCCGGTGACATCAA-3′	86
Smooth muscle actin alpha 2 (ACTA2)	Forward: 5′- GACAATGGCTCTGGGCTCTGTAA-3′ Reverse: 5′-CTGTGCTTCGTCACCCACGTA-3′	147
Glyceraldehyde 3-phosphate dehydrogenase (GAPDH)	Forward: 5′-GACCACTTTGTCAAGCTCATTTCC-3′ Reverse: 5′-GTGAGGGTCTCTCTCTTCCTCTTGT-3′	151

### Collagen gel contraction assay

CCC-ESF-1 contractility was determined using collagen gel contraction assay. Cells were pretreated with baicalein for 2 h before adding TGF-β1 or PDGF and then mixed with the neutralized rat-tail collagen type I solution (pH 7.4, final concentration of collagen at 1 mg/mL) at a final cell density of 4 × 10^5^ cells/mL. The mixture of cells in collagen (500 μL) was added to a 24-well plate and polymerized at room temperature. After detaching the gel, the cell-collagen mixture was treated with media containing baicalein and TGF-β1 or PDGF for 48 h. Gel images were taken and the contraction was measured by analyzing the gel area using ImageJ.

### Mouse model of SSc

Female C57BL/6 J mice (6–7 weeks) were obtained from Beijing Vital Laboratory Animal Technology (Beijing, China). They were maintained in a specific pathogen-free laboratory with food and water ad libitum. The study protocol was approved (20162032) by the Animal Ethical and Welfare Committee of Institute of Chinese Materia Medica, China Academy of Chinese Medical Sciences. Animal procedures were in accordance with the Institutional Animal Care and Use Committee guidelines.

The bleomycin-induced model of skin fibrosis was described by Yamamoto and colleagues [32]. Bleomycin (Nippon Kayaku, Tokyo, Japan) was dissolved in physiologic 0.9% NaCl (1 mg/mL) and sterilized by filtration. Mice were divided randomly into six groups with ≥ 9 mice in each group. After shaving of the back, bleomycin (100 μL) or 0.9% NaCl (100 μL) was injected (s.c.) into well-defined areas (1 cm^2^) once daily, along with oral gavage with vehicle control (0.5% carboxymethylcellulose sodium), D-penicillamine (125 mg/kg; positive control) or baicalein (25, 50, or 100 mg/kg) once daily for 4 weeks.

### Histology and fibrosis quantification in mice

After killing, the injected skin areas were fixed in 4% paraformaldehyde in PBS, embedded in paraffin, and stained with hematoxylin and eosin (H&E; to demonstrate the general morphology of tissue) or Masson’s trichrome (MT; to evaluate the collagen-based matrix). Dermal thickness was determined by measuring the mean distance between the epidermal–dermal junction and dermal–subcutaneous-fat junction. Inflammatory infiltrates were evaluated on H&E-stained sections according to a semiquantitative method using a grading score of inflammation severity as described by Gallet and colleagues [33]. The collagen content in lesional skin was analyzed by the hydroxyproline assay according to manufacturer (Nanjing Jiancheng Bioengineering Institute, Nanjing, China) instructions, and is expressed as μg hydroxyproline/wet weight of tissue (mg).

### ELISAs for autoantibodies and cytokines in serum

Mice were anesthetized with pentobarbital sodium (30 mg/kg, i.v.). Blood was collected from the retro-orbital venous sinus. Serum was separated by centrifugation at 3500 × *g* for 20 min at room temperature, collected into a tube, and stored at − 80 °C. Serum levels of anti-scleroderma 70 (Scl-70), anti-double stranded DNA (dsDNA), anti-centromeres and anti-polymyositis-scleroderma (PM-Scl) were measured using ELISA kits according to manufacturer (Euroimmun Medizinische Labordiagnostika, Luebeck, Germany) instructions with minor modifications. A serum dilution of 1:5 was used for the determination of all autoantibodies. The secondary antibody was goat polyclonal anti-mouse IgG H&L conjugated to horseradish peroxidase (Abcam, Cambridge, UK). The OD was measured at 450/650 nm. Serum levels of IL-1β, IL-2, IL-4, IL-6, IL-17A, TNF-α, MCP-1, and macrophage inflammatory protein-1 beta (MIP-1β) were measured using MILLIPLEX® MAP kits (Merck Millipore, Whitehouse Station, NJ, USA) according to manufacturer instructions. Serum level of IL-11 was measured using an ELISA kit according to the manufacturer’s instructions (Elabscience, Wuhan, China).

### Flow cytometry

Single-cell suspensions were obtained from mouse spleens in PBS. Erythrocytes were lysed with Red Blood Cell lysis buffer. Spleen cells were labeled with monoclonal antibodies specific for cell-surface markers B220 (PE-Cy7, RA3-6B2) from BD Pharmingen (San Diego, CA, USA) and CD27 (PE, LG.3A10) from Miltenyi Biotec (Bergisch Gladbach, Germany). Samples were assessed by the CytoFLEX™ flow cytometry system (Beckman Coulter, Fullerton, CA, USA). At least 30,000 lymphocyte-gated cells were collected routinely for each sample. B cells were identified as B220^+^ lymphocytes. Memory B cells were identified as CD27^+^B220^+^ lymphocytes.

### Protein extraction and western blotting

The total protein of the skin was prepared in Cell Lysis Buffer plus 1 mM phenylmethanesulfonyl fluoride (Cell Signaling Technology, Danvers, MA, USA). Tissues were homogenized with 2 metal beads at a speed of 60 Hz for 2 min. After centrifugation at 12,000 × g for 20 min at 4 °C, the protein concentration was measured by the Bradford method, western blotting was done as described previously [9]. The primary antibodies for TGF-β1, α-SMA, COL1A1, phosphor-SMAD3, SMAD3, phospho-ERK1/2 (Thr202/Tyr204) (p-ERK), ERK1/2 and GAPDH were obtained from Cell Signaling Technology (Danvers, MA, USA).

### Statistical analyses

Data are the mean ± SD (in vitro) or mean ± SEM (in vivo). Statistical analyses were undertaken using Prism 8.0.2 (GraphPad, La Jolla, CA, USA). Significant differences of multiple groups were determined by ANOVA followed by Dunnett test or Kruskal–Wallis test for post hoc comparisons. *P* < 0.05 was considered significant.

## Results

### Baicalein attenuates TGF-β1- and PDGF-induced fibrosis in dermal fibroblasts

**Fig. 1:**
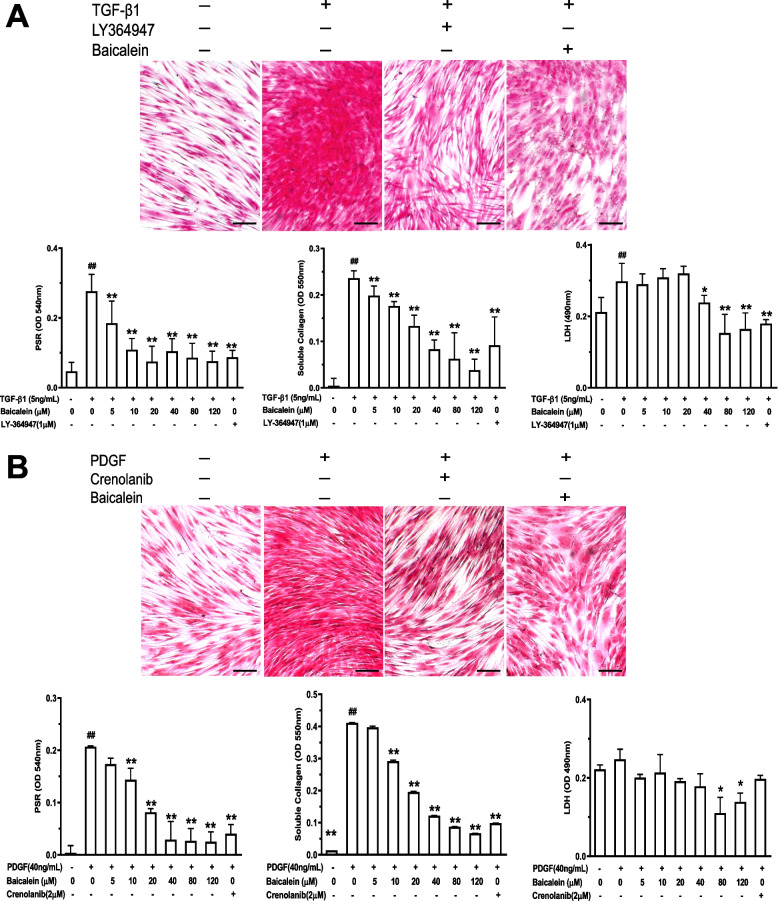
Baicalein inhibits TGF-β1- (**A**) or PDGF (**B**)-induced fibrosis in CCC-ESF-1 cells. Cells were seeded in collagen I-coated plates and treated/not treated with TGFβ1 (5 ng/mL) or PDGF (40 ng/mL) in the presence or absence of baicalein (5–120 μM) for 48 h. Total collagen deposition was visualized by PSR staining and quantified by spectrophotometry. Soluble collagen in the supernatant was detected by the Sircol Soluble Collagen Assay. Cytotoxicity was assessed using the lactate dehydrogenase release (LDH) assay. Representative images were shown, scale bar = 200 μm. Quantitative data were the mean ± SD from one representative experiment out of ≥ 3 experiments with similar results, n ≥ 4 wells per group. ^#^*P* < 0.05, ^##^*P* < 0.01 *vs*. no TGF-β1 or PDGF group; *P < 0.05, **P < 0.01 *vs*. TGF-β1- or PDGF-only group

As proof of the principle that TGF-β1 and PDGF are the central mediators of tissue fibrosis in SSc [19, 34], we selected these two stimulators to establish in vitro models to analyze the antifibrotic activity of baicalein. The total deposition of collagen, soluble collagen in supernatants, and toxicity to CCC-ESF-1 cells were analyzed. Addition of TGF-β1 (5 ng/mL) or PDGF (40 ng/mL) induced a potent accumulation of total collagen and collagen secretion (Fig. 1). Baicalein could abrogate the deposition of total collagen significantly, with a 50% effective concentration (EC50) of 18.73 ± 3.02 μM in the TGF-β1-induced model and 18.07 ± 2.67 μM in the PDGF-induced model. Baicalein could reduce the secretion of soluble collagen markedly, with an EC50 of 27.90 ± 5.86 μM in the TGF-β1-induced model and 22.54 ± 5.83 μM in the PDGF-induced model. The LDH assay showed that exposure to baicalein (5–120 μM) resulted in relatively low toxicity against TGF-β1- or PDGF-treated CCC-ESF-1 cells.

**Fig. 2:**
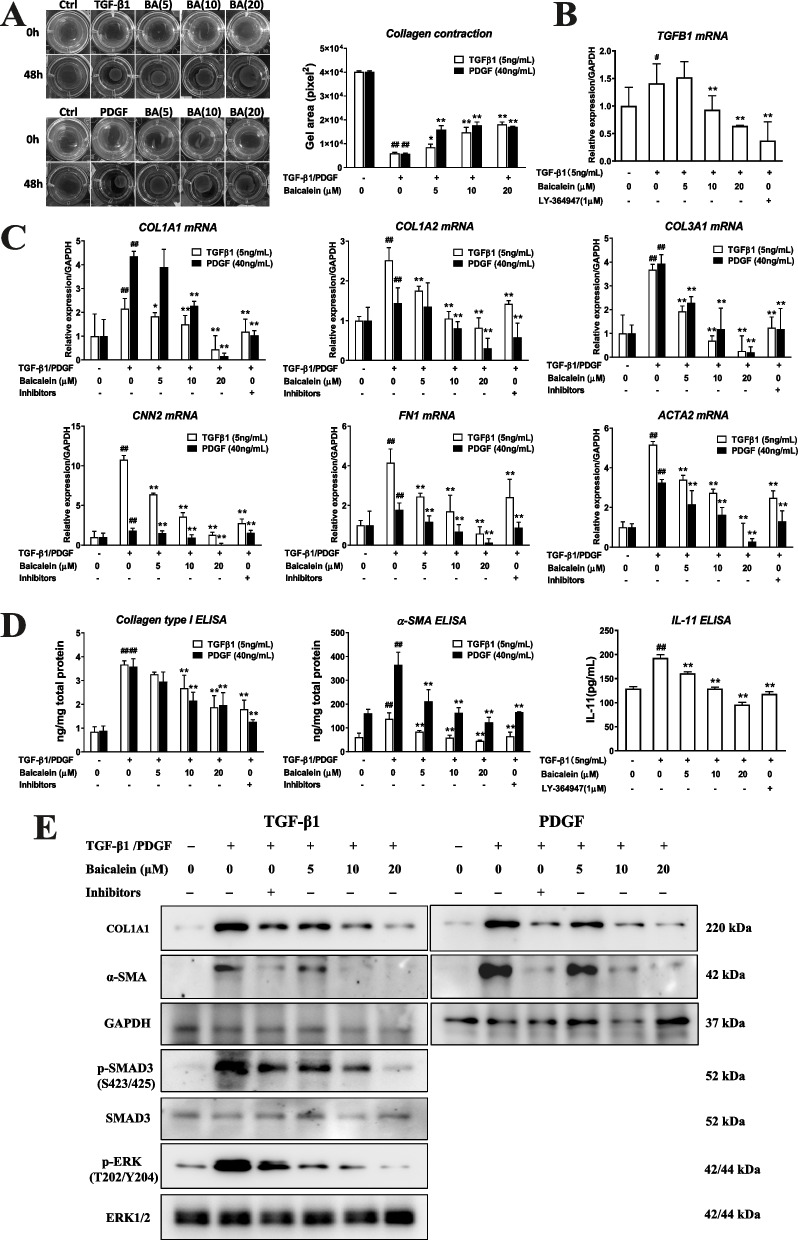
Baicalein suppresses the expression of fibrogenesis markers and TGF-β1 signaling pathway in dermal fibroblasts. Cells were treated with TGFβ1 (5 ng/mL) or PDGF (40 ng/mL) in the presence or absence of baicalein (5–120 μM) for 48 h, and collected for the collagen contractibility of dermal fibroblasts by collagen gel contraction assay (**A**), RT-qPCR (**B**, **C**), ELISAs (**D**) and western blotting (**E**). Quantitative data were the mean ± SD of 3–5 replicates. Each experiment was repeated thrice. ^#^*P* < 0.05, ^##^*P* < 0.01 *vs*. no TGF-β1 or PDGF group; *P < 0.05, **P < 0.01 *vs*. TGF-β1- or PDGF-only group

We undertook collagen gel contraction assay to measure the contractility of dermal fibroblasts. As shown in Fig. 2A, TGF-β1 and PDGF significantly increased the collagen contraction capability compared to the vehicle control, and this effect was potently suppressed by treatment with baicalein (5–20 μM).

Moreover, the expression of molecular markers contributing to collagen deposition and myofibroblast differentiation in the fibrogenic process was analyzed by RT-qRCR, ELISAs and western blotting. In the TGF-β1-induced model, treatment with baicalein (5–20 μM) resulted in dose-dependent downregulation of the gene expression of fibrotic-related markers, including collagen type I alpha 1 chain (COL1A1), COL1A2, COL3A1, cellular communication network factor 2 (CNN2), fibronectin 1 (FN1), TGFB1, and smooth muscle actin alpha 2 (ACTA2) (Fig. 2B–C), as well as protein expression of type-I collagen and α-SMA (Fig. 2D–E). Similar results were observed in PDGF-induced cells: baicalein potently inhibited the stimulatory effects of PDGF on all the markers of fibrogenesis in a dose-dependent manner (Fig. 2C–E).

### Baicalein ameliorates bleomycin-induced altered skin architecture and inhibits skin fibrosis

**Fig. 3:**
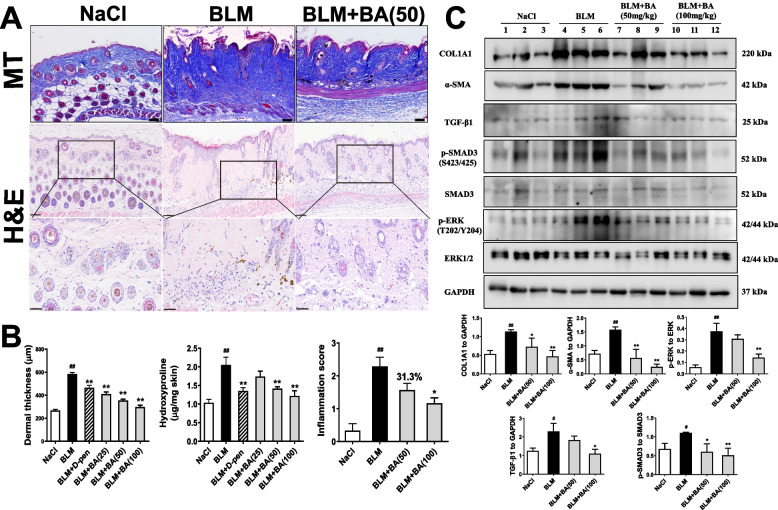
Baicalein attenuates fibrosis in mice suffering from bleomycin-induced SSc. Bleomycin (1 mg/mL; 100 μL/mice) was injected subcutaneously into the back of mice once daily for 4 weeks. Mice underwent oral gavage with vehicle control (0.5% carboxymethylcellulose sodium), D-penicillamine (125 mg/kg), or baicalein (BA, 25–100 mg/kg) once daily for 4 weeks. Representative images of the H&E and Masson’s-trichrome staining of the skin of mice in each group were shown. In H&E, scale bar = 100 μm (upper) and scale bar = 50 μm (lower). In Masson’s-trichrome, scale bar = 100 μm (**A**). Dermal thickness (*n* = 9), hydroxyproline content (*n* = 9) and inflammation score (*n* = 6–7) were quantified (**B**). **C** protein from lesional skins was analyzed by western blotting for COL1A1, α-SMA, TGF-β1/SMAD3 activation and ERK activation in randomly selected mice of each group. Levels of expression were measured by densitometry and expressed as normalized density (*n* = 3–4). Representative immunoblots for 3 mice of each group were shown. Quantitative data were the mean ± SEM. ^#^*P* < 0.05, ^##^*P* < 0.01 *vs*. NaCl-treated mice. * *P* < 0.05, ** *P* < 0.01 *vs*. bleomycin (BLM)-treated mice

Given that baicalein inhibited the deposition and secretion of collagen in vitro, we investigated the effect of baicalein on dermal fibrosis in a bleomycin-induced fibrosis model in mice. We undertook H&E staining, MT staining, the hydroxyproline assay and western blotting towards this aim. Compared with NaCl-treated mice, subcutaneous injection with bleomycin significantly induced severe disruption of dermal architecture, cutaneous fibrosis (as represented by increased dermal thickening), ECM deposition, and marked accumulation of hydroxyproline content (*P* < 0.05) (Fig. 3A, B). Western blotting showed significantly increased protein expression of type-I collagen and α-SMA in the skin (Fig. 3C). Mice administered baicalein (25–100 mg/kg) had restored dermal architecture, significantly decreased dermal thickness, and ameliorated excessive collagen accumulation in a dose-dependent manner (Fig. 3A, B). According to western blotting, baicalein (50 and 100 mg/kg) resulted in a marked down-regulation of expression of type-I collagen and α-SMA (Fig. 3B). Taken together, these data indicated the antifibrotic effect of baicalein in vivo.

### Baicalein suppresses TGF-β-mediated activation in dermal fibrosis

In SSc, TGF-β1-dependent SMAD phosphorylation is considered to be an essential signaling pathway in dermal fibrosis [19]. We first detected the effect of baicalein on TGF-β signaling for skin fibrosis in dermal fibroblasts using RT-qPCR or western blotting for expression of TGF-β1 and activation of SMAD3. Treatment with TGF-β1 (5 ng/mL) resulted in a notable increase of mRNA expression of endogenous TGF-β1 (Fig. 2B) and enhanced phosphorylation of SMAD3 (Fig. 2E) in CCC-ESF-1 cells. Baicalein (5–20 μM) brought down the gene expression of TGF-β1 and protein expression of phosphorylated SMAD3 (Fig. 2B, E). Bleomycin-induced scleroderma has been featured by aberrant activation of TGF-β1 and led to SMAD-dependent fibrosis [32]. We further investigated the effect of baicalein on the SMAD3-dependent pathway in bleomycin-treated mice (Fig. 3C). Compared with NaCl-treated mice, bleomycin-treated mice showed significant increase of protein expression of TGF-β1, as well as the enhanced phosphorylation of SMAD3. Baicalein markedly inhibited bleomycin-induced expression of TGF-β1 and phosphorylated SMAD3 in a dose-dependent manner. These results showed that TGF-β1/SMAD3 pathway might be involved in the antifibrotic efficacy of baicalein.

Previous study has also suggested the role of IL-11, a profibrotic cytokine, in TGF-β-driven fibroblast activation due to SSc [35–37]. IL-11-dependent extracellular signal-regulated kinase (ERK) cascade is required for the profibrotic responses induced by TGF-β in dermal fibroblasts [35]. Here, we tested the efficacy of baicalein on inhibiting IL-11-mediated ERK1/2 phosphorylation from TGF-β1-induced dermal fibroblasts. Compared to no TGF-β1 control, TGF-β1 stimulation resulted in the upregulated expression of IL-11 and the activation of ERK1/2 in CCC-ESF-1 cells. Baicalein could suppress TGF-β1-induced IL-11 expression and ERK1/2 phosphorylation in a dose-dependent manner (Fig. 2D, E). In bleomycin-induced mice, circulating IL-11 levels were significantly elevated in the serum compared with the NaCl-treated control. Baicalein treatment dose-dependently reduced the serum level of IL-11 induced by bleomycin and downregulated the expression of phosphorylated ERK1/2 in the skin of bleomycin-treated mice (Fig. 3). These data suggested that IL-11 signaling might play a role, at least in part, in the inhibition of baicalein on TGF-β-driven fibroblast activation in dermal fibrosis of SSc.

### Baicalein attenuates bleomycin-induced inflammation

SSc pathogenesis is closely associated with inflammatory abnormalities in the initial phase of fibrosis [38]. Bleomycin-induced SSc involves dysregulation of levels of proinflammatory cytokines [19, 39]. We used histopathology to evaluate the infiltration of inflammatory cells in dermal-tissue sections, and ELISAs to measure serum levels of cytokines and chemokines. Subcutaneous injection of bleomycin resulted in moderate-to-severe inflammation in dermal tissue because of the recruitment of inflammatory cells (macrophages, lymphocytes, and neutrophils). Baicalein treatment revealed a modest number of inflammatory infiltrates and markedly decreased the score of inflammatory grade (Fig. 3A, B).

**Fig. 4:**
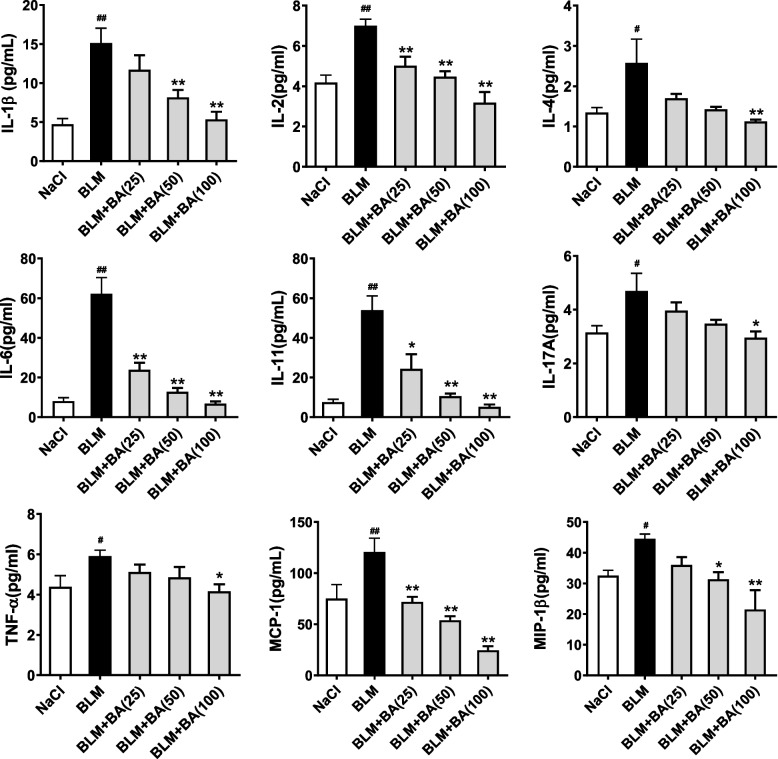
Baicalein reduces serum levels of cytokines and chemokines in mice with bleomycin-induced SSc. Bleomycin (1 mg/mL; 100 μL/mice) was injected subcutaneously into the back of mice once daily for 4 weeks. Mice were oral gavage with vehicle control (0.5% carboxymethylcellulose sodium) or baicalein (BA, 25–100 mg/kg) once daily for 4 weeks. Serum was collected for cytokine/chemokine analysis using MILLIPLEX™ MAP. Quantitative data were the mean ± SEM from 7–10 mice in each group. ^#^*P* < 0.05, ^##^*P* < 0.01 *vs*. NaCl-treated mice. * *P* < 0.05, ** *P* < 0.01 *vs*. bleomycin (BLM)-treated mice

In accordance with the data from H&E staining, bleomycin administration increased serum levels of IL-1β, IL-2, IL-4, IL6, IL-17A, TNF-α, MCP-1, and MIP-1β dramatically (*P* < 0.05). Significant reduction of serum levels due to baicalein treatment was observed for all of these eight cytokine/chemokine markers in a dose-dependent manner (*P* < 0.05) (Fig. 4).

### Baicalein inhibits bleomycin-induced autoantibody overproduction in serum

**Fig. 5:**
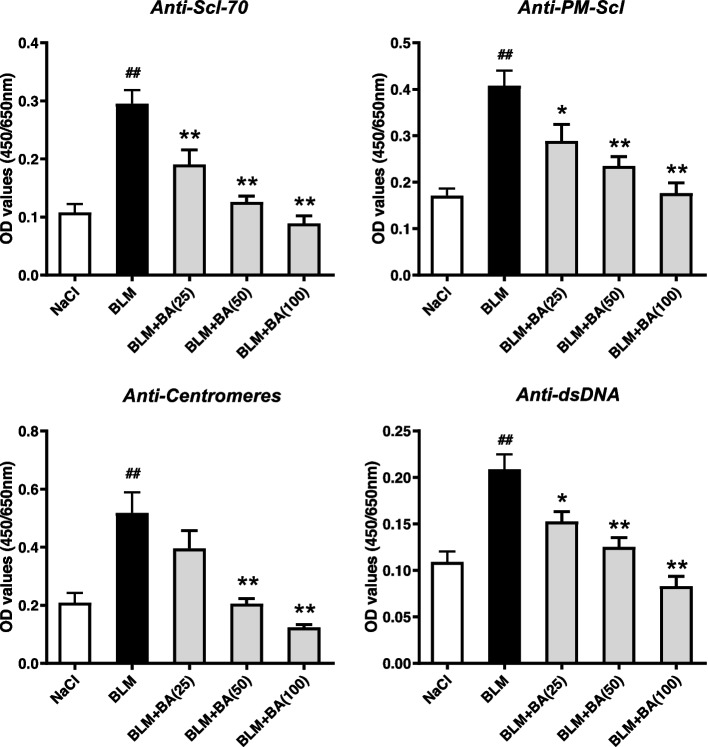
Baicalein inhibits bleomycin-induced autoantibody overproduction in serum. Quantitative data were the mean ± SEM from 7–10 mice in each group. ^#^*P* < 0.05, ^##^*P* < 0.01 *vs*. NaCl-treated mice. * *P* < 0.05, ** *P* < 0.01 *vs*. bleomycin (BLM)-treated mice

The presence of autoantibodies is a central feature of immune activation in human SSc [40]. Production of specific antibodies, such as anti-nuclear and anti-centromere autoantibodies, is detectable and associated with pathologic processes in a bleomycin-induced model of SSc in mice [41]. To elucidate if baicalein affects autoantibody secretion, we measured autoantibody levels in the serum of mice given bleomycin. Baicalein reduced the serum levels of anti-Scl-70, anti-PM-Scl, anti-centromeres, and anti-dsDNA in a dose-dependent manner compared with those in bleomycin-induced mice (Fig. 5).

### Baicalein modulates bleomycin-induced alteration of B-cell distribution

**Fig. 6:**
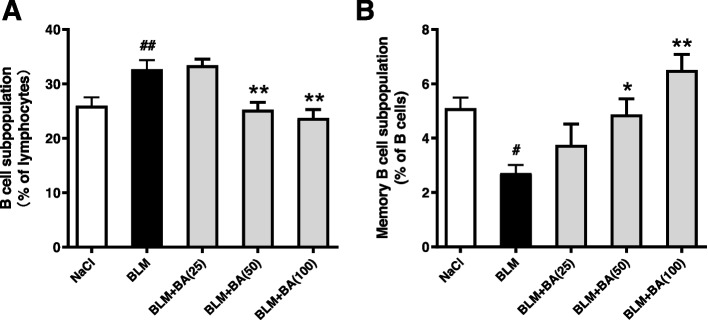
Baicalein inhibits abnormalities of B-cell distribution in a bleomycin-induced SSc model in mice. Mice were treated for 4 weeks. Spleen cells were collected to calculate the proportion of B cells (**A**) and CD27^+^ memory B cells (**B**). Quantitative data were the mean ± SEM from 9 mice in each group. ^#^*P* < 0.05, ^##^*P* < 0.01 *vs*. NaCl-treated mice. * *P* < 0.05, ** *P* < 0.01 *vs*. bleomycin (BLM)-treated mice

Abnormalities in the homeostasis of B cells are thought to play an essential part in SSc [23]. We calculated the percentage of B cells and memory B cells in mouse spleens. Bleomycin-treated mice exhibited a potent increase in the B-cell subpopulation (B220^+^), whereas the subpopulation of memory B cells (B220^+^CD27^+^) was attenuated markedly, in mouse spleens (Fig. 6). Compared with bleomycin treatment only, exposure to baicalein markedly reduced the proportion of B cells to lymphocytes and increased the proportion of memory B cells to B cells in a dose-dependent manner.

## Discussion

SSc is a systemic autoimmune disorder featured by an early inflammatory reaction followed by diffuse fibrosis and vascular changes in the skin and organs. Progressive fibrosis caused by excessive accumulation of the ECM and collagen is a hallmark of SSc [1, 19]. TGF-β1 [19] and PDGF [34] are the central mediators of tissue fibrosis in SSc. They induce collagen deposition as well as the proliferation, migration, accumulation, and activation of mesenchymal cells. Several studies have reported that baicalein has therapeutic effects on fibrosis (pulmonary, hepatic, cardiac, and renal interstitial) [8–11]. Baicalein (150 μM) has been reported to suppress TGF-β1-induced collagen deposition as well as the expression of type-I collagen and α-SMA by activating the p38 mitogen-activated protein kinase/c-Jun N-terminal kinase pathway in NIH-3T3 cells [14]. Baicalein has also been found to attenuate the production of type-I collagen, downregulate expression of CNN2 and ACTA2, and retard the formation of α-SMA filaments in TGF-β1-stimulated human lung fibroblasts [8, 12]. In renal fibroblasts, baicalein (20–80 μM) has been shown to specifically inhibit TGF-β1-induced ECM accumulation as well as mRNA and protein expression of type-I collagen [9]. In accordance with those observations, we found that after treatment with baicalein (5–120 μM) for 48 h, TGF-β1-induced accumulation and secretion of collagen in skin fibroblasts was attenuated significantly in a dose-dependent manner (Fig. 1). In addition, baicalein could abrogate the enhanced collagen contraction potential in TGF-β1-treated fibroblasts markedly. RT-qPCR and ELISAs showed that incubation with baicalein resulted in fairly potent suppression of mRNA expression of fibrogenic markers (COL1A1, COL1A2, COL3A1, CCN2, FN1, TGFB1, and ACTA2), as well as the decreased protein expression of type-I collagen and α-SMA (Fig. 2). In agreement with the observations from the TGF-β1-induced model, treatment of PDGF-induced CCC-ESF-1 cells with baicalein under identical conditions showed a similar efficacious trend against fibrosis, as evident from reduced collagen release and deposition, deregulation of fibrogenesis-related mRNA expression, and suppressed fibroblast activation with decreased α-SMA expression and reduced contractile activity (Figs. 1, and 2). These results suggest that baicalein has direct antifibrotic activity because it specifically inhibits TGF-β1- and PDGF-mediated ECM accumulation and fibroblast activation.

After documenting the in vitro findings stated above, the in vivo efficacy of baicalein against SSc progression was investigated. It has been reported that baicalein (50 and 100 mg/kg) treatment can ameliorate kidney fibrosis significantly in mice with unilateral ureteral obstruction [13]. Administration of baicalein (25 and 100 mg/kg) has been shown to reverse angiotensin II-induced and aortic banding-induced cardiac fibrosis [26, 42]. Baicalein (50 and 100 mg/kg) has been shown to attenuate bleomycin-induced pulmonary fibrosis [43]. In the present study, we used the widely accepted bleomycin-induced experimental model of skin fibrosis to determine the antifibrotic effect of baicalein on SSc. We found that treatment with baicalein (25–100 mg/kg) inhibited bleomycin-induced skin fibrosis (as evident from reduced dermal thickness, ameliorated excessive collagen accumulation with decreased hydroxyproline content and protein expression of type-I collagen, as well as inhibited myofibroblast differentiation) in a dose-dependent manner (Fig. 3).

TGF-β1 has long been identified to play a fundamental role in SSc pathogenesis through both SMAD-dependent and SMAD-independent pathways. Activated TGF-β1 stimulates constitutive SMAD2/3 phosphorylation and nuclear translocation, thereby initiates and sustains fibrotic events in SSc. Thus, TGF-β1/SMAD pathway is an effective target for the treatment of SSc [19]. Baicalein has been found to suppress endogenous TGF-β1 expression and activation of SMAD3 in kidney interstitial fibroblast NRK-49F cells [9]. Our data demonstrated that baicalein could downregulate the expression of TGF-β1 and inhibit the phosphorylation of SMAD3 in both TGF -β1 induced dermal fibroblasts and skins of bleomycin-induced mice of SSc, thus might result in decreased expression of fibrogenic markers, as shown in our findings stated above (Figs. 2, and 3). Apart from the TGF-β/SMAD3 signaling, non-canonical TGF-β signaling acts through IL-11-dependent pathway to elicit myofibroblast activation in some tissues [44]. IL-11 has recently been recognized as a TGF-β-responsive profibrotic cytokine and was shown to be highly upregulated in dermal fibroblasts from SSc skin and in pulmonary fibroblasts from patients suffering from interstitial lung disease associated with SSc [36, 37]. A previous study found that inhibition of IL-11 signaling using a neutralizing IL-11 antibody, siRNA against IL-11RA or MEK inhibitor U0126 blocked the fibrotic effects of TGF-β, including cell proliferation, matrix production, and cell migration, in human dermal fibroblasts [35]. In keeping with data on human dermal fibroblasts, we observed that TGF-β1 consistently induced IL-11 upregulation concomitant with ERK1/2 phosphorylation in dermal CCC-ESF-1 fibroblasts. Bleomycin-induced fibrosis mouse model also demonstrated the increased level of IL-11 in serum and ERK1/2 phosphorylation in the skin. These results are in agreement with previous studies that IL-11-mediated ERK1/2 cascade plays a central role for the SSc fibroblast activation induced by TGF-β in vitro and skin fibrosis in the mouse model [35, 45, 46]. In current work, baicalein treatment was found to significantly reduce the expression of IL-11 and inhibit ERK1/2 activation both in TGF-β1-stimulated dermal fibroblasts and bleomycin-induced mice. These findings suggested that SMAD3 signaling and IL-11-dependent ERK1/2 signaling might be associated with the inhibitory effect of baicalein on TGF-β1-induced fibrosis in vitro and skin fibrosis in bleomycin-induced mice. Further studies are required to explain the role of canonical TGF-β/SMAD signaling and non-canonical TGF-β and IL-11 signaling in the fibrosis of SSc.

Immunologic dysregulation (autoimmunity, infiltration, and activation of immune cells) is an essential feature of SSc [19, 20]. B cells are involved directly and indirectly in SSc pathogenesis through the production of autoantibodies and release of proinflammatory/ profibrotic cytokines and chemokines [19, 21]. Previous studies have reported that SSc patients had altered B-cell homeostasis and activation of B cells, with increased numbers of B cells but decreased percentage and absolute counts of memory B cells [23–25]. Several types of antinuclear antibodies (e.g., topoisomerase I, RNA polymerase III, ribonuclear protein U1, centromeres, and nucleolar antigen PM/Scl) were detected in 43% of SSc patients [40, 47]. In our mouse model, subcutaneous injection of bleomycin resulted in an increased B-cell subpopulation and reduced percentage of CD27^+^ memory B cells, which were concomitant with autoantibody production (Figs. 5, and 6). These results suggested that B cells might be hyperactivated and had important roles in the pathogenesis in bleomycin-induced mice, and these data were comparable with the results from studies employing other mice models of SSc [48–50]. Baicalein treatment potently attenuated the serum levels of autoantibodies produced by B cells in bleomycin-induced mice (Fig. 5). Under identical conditions, the percentage of splenic B cells was reduced and the percentage of CD27^+^ memory B cells was increased, which suggested that baicalein ameliorated the immunologic state induced by bleomycin through regulating the distribution of B cells (Fig. 6).

Considering that inflammation occurs in the early stage of fibrosis and is strongly responsible for the development of SSc [38], we measure the anti-inflammatory efficacy of baicalein in bleomycin-induced SSc. Some pro-inflammatory/inflammatory cytokines and chemokines, such as IL-4, IL-6, IL-10, MCP-1, and MIP-1, are at high levels in the blood, skin, and lungs. Their levels have been reported to correlate with distinct clinical features and disease severity [51]. These mediators have also been identified as fibrogenic markers because they activate fibroblasts and enhance the production of collagen and the ECM in fibrosis pathogenesis in some mouse models of SSc [39, 41, 52]. In the present study, bleomycin injection led to overexpression of pro-inflammatory/inflammatory cytokines and chemokines, while baicalein treatment significantly attenuated the serum levels of cytokines and chemokines in mice (Fig. 4). In parallel, we detected inflammatory infiltration in the skins of bleomycin-induced mice. Baicalein treatment markedly alleviated the number of inflammatory or immunocytes (neutrophils, macrophages, or lymphocytes) infiltrated into the skin (Fig. 3A), which occurred in accordance with the reduced serum levels of cytokines and chemokines. Previous works describing the role of interaction between dermal fibroblasts and immune cells in SSc fibrosis have demonstrated that B lymphocytes [53] and macrophages [54] from SSc patients could produce IL-6 and TGF-β to activate fibroblasts and produce collagen [4, 21]. Thus, we think that besides the direct effect on TGF-β1 pathway as mentioned above, baicalein might inhibit this pathway indirectly by ameliorating the inflammatory infiltrates.

Our data demonstrate that baicalein exerts dose-dependently anti-fibrotic effects against SSc by suppressing TGF-β1- and PDGF-mediated ECM accumulation in vitro as well as alleviating fibrosis, regulating B-cell abnormalities, and attenuating inflammation in bleomycin-induced scleroderma in vivo. Baicalein has a potential to be developed as a therapeutic candidate against SSc.

## Supplementary Information

**Additional file 1.**

## Data Availability

All data generated or analyzed during this study are included in this published article [and its supplementary information files].
