# Supplementary material for: Baicalein alleviates fibrosis and inflammation in systemic sclerosis by regulating B-cell abnormalities
Source: BMC Complement Med Ther. 2023 Feb 21;23:62. doi: 10.1186/s12906-023-03885-1 (PMC9942410; doi:10.1186/s12906-023-03885-1)
Supplement: Supplementary file 1 — Additional file 1. [file 12906_2023_3885_MOESM1_ESM.docx]

**Supplementary Information**

**Supplementary Figure 2. Original western blotting for TGF-β1/SMAD3 and ERK pathways in TGF-β1- and PDGF-induced dermal fibroblasts**

**
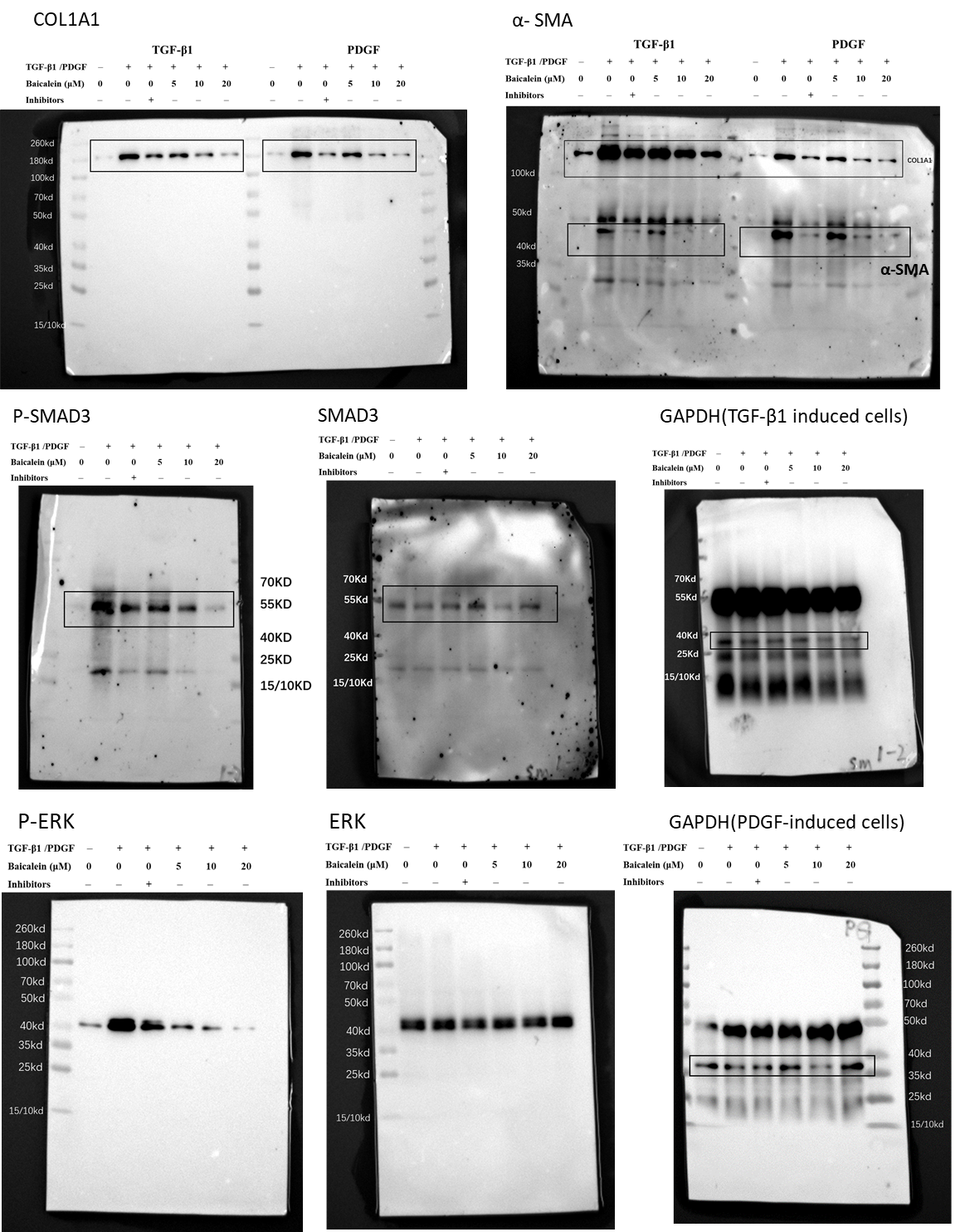
**

**Supplementary Figure 3. Original western blotting for protein from lesional skins in bleomycin-induced mice.** From left to right Lane1-3: NaCl control, Lane 4-6: BLM, Lane 7-9: BLM+baicalein (50mg/kg), Lane 10-12: BLM+baicalein (100mg/kg).

**
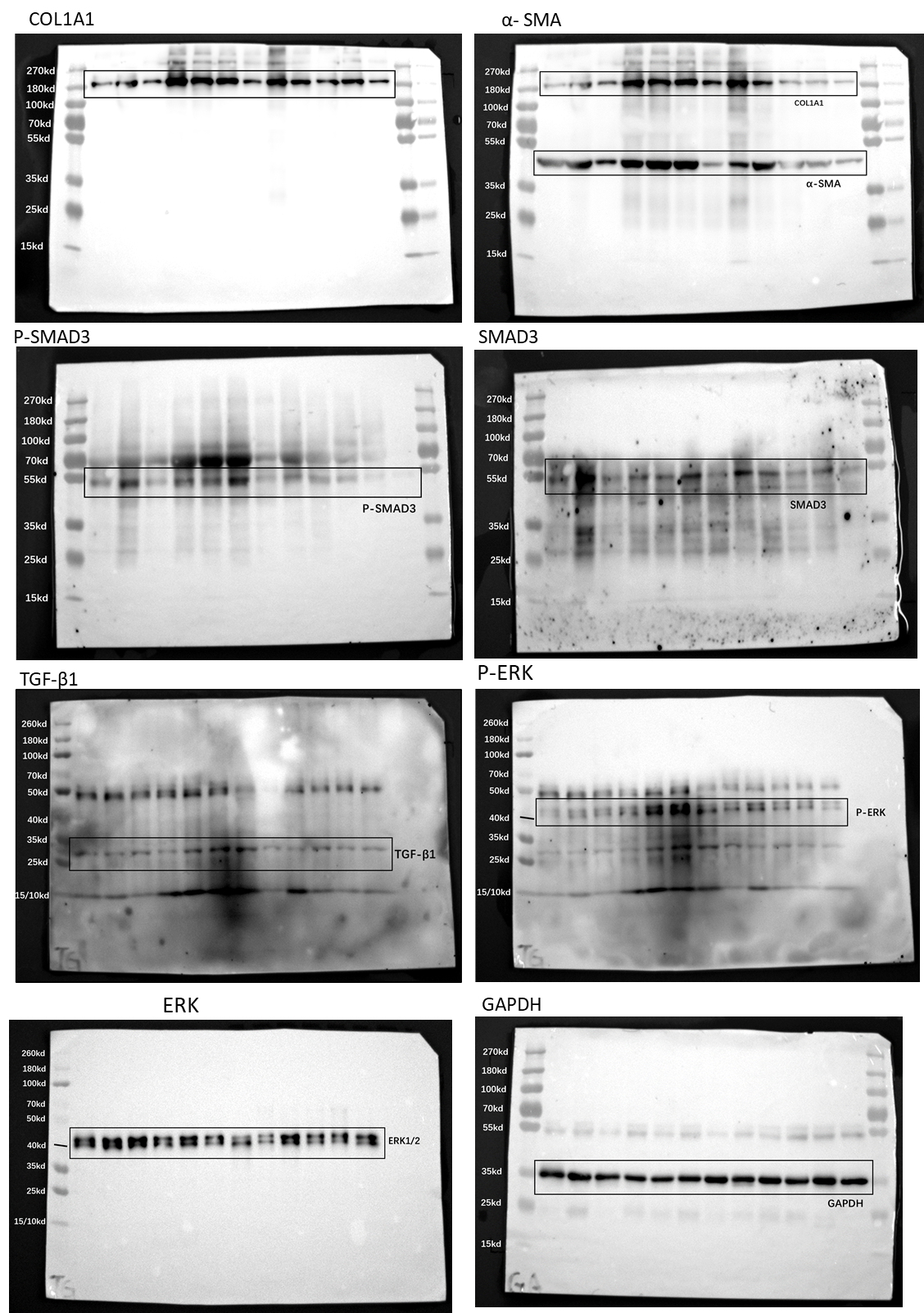
**
